# Polyamine-mediated mechanisms contribute to oxidative stress tolerance in *Pseudomonas syringae*

**DOI:** 10.1038/s41598-023-31239-x

**Published:** 2023-03-15

**Authors:** Leandro Solmi, Franco R. Rossi, Fernando. M. Romero, Marcel Bach-Pages, Gail M. Preston, Oscar A. Ruiz, Andrés Gárriz

**Affiliations:** 1 Instituto Tecnológico de Chascomús (CONICET-UNSAM), Avenida Intendente Marino Km 8.2, Chascomús, CP7130 Buenos Aires, Argentina; 2 Escuela de Bio y Nanotecnologías (UNSAM), Buenos Aires, Argentina; 3 Department of Plant Sciences, University of Oxford, Oxford, UK

**Keywords:** Microbiology, Biochemistry

## Abstract

Bacterial phytopathogens living on the surface or within plant tissues may experience oxidative stress because of the triggered plant defense responses. Although it has been suggested that polyamines can defend bacteria from this stress, the mechanism behind this action is not entirely understood. In this study, we investigated the effects of oxidative stress on the polyamine homeostasis of the plant pathogen *Pseudomonas syringae* and the functions of these compounds in bacterial stress tolerance. We demonstrated that bacteria respond to H_2_O_2_ by increasing the external levels of the polyamine putrescine while maintaining the inner concentrations of this compound as well as the analogue amine spermidine. In line with this, adding exogenous putrescine to media increased bacterial tolerance to H_2_O_2_. Deletion of arginine decarboxylase (*speA*) and ornithine decarboxylate (*speC*), prevented the synthesis of putrescine and augmented susceptibility to H_2_O_2_, whereas targeting spermidine synthesis alone through deletion of spermidine synthase (*speE*) increased the level of extracellular putrescine and enhanced H_2_O_2_ tolerance. Further research demonstrated that the increased tolerance of the Δs*peE* mutant correlated with higher expression of H_2_O_2_-degrading catalases and enhanced outer cell membrane stability. Thus, this work demonstrates previously unrecognized connections between bacterial defense mechanisms against oxidative stress and the polyamine metabolism.

## Introduction

Higher organisms respond to potentially harmful microbes with the production of an arrange of molecules known as reactive oxygen species (ROS), such as superoxide (O_2_^·−^), the hydroxyl radical (OH^.^) and hydrogen peroxide (H_2_O_2_). For instance, ROS synthesis is a well-known defense response evoked in plants after pathogen detection, which consists in the rapid synthesis and buildup of high concentrations of these molecules in the vicinity of the invading microbe. This response creates an oxidative environment that directly and indirectly acts to impede microbial growth and disease development^1^. Oxidative stress in microbes may also be caused by the action of plant produced antimicrobial compounds, which promote higher rates of internal ROS production^2,3^. As a result, microorganisms must have mechanisms to deal with ROS generated from many sources. If this is not properly managed, it can have negative consequences on growth and development due to the oxidative damage of important biomolecules^4^.

A variety of defense mechanisms to counteract oxidative stress have been developed in bacteria^5–7^. The coordination of such defensive systems enables cells to survive against oxidative stress, even though their tolerance depends on the type of the mechanisms being deployed, the concentration of ROS to deal with, and the length of exposure. For instance, it has been shown that cells are protected by extracellular polysaccharides that are secreted or remain attached to membranes^7,8^, and that Gram-negative bacteria increase outer-membrane stability and modify its permeability as survival strategies^9,10^. Other important antioxidant mechanisms in these microbes rely on the activities of a wide range of ROS detoxifying enzymes, the production of antioxidative molecules, and metabolic remodeling^11^. The main enzymes degrading ROS include the superoxide dismutases which produce hydrogen peroxide (H_2_O_2_) from superoxide radical, as well as catalases (Kat) and peroxidases that reduce H_2_O_2_ to water. In turn, the synthesis of antioxidant molecules such as glutathione plays an essential function as it maintains the intracellular redox state and regulates post translationally the function of different key proteins required for the antioxidant response^12^. At last, adaptation of metabolism may retard the damages caused by oxidative stress. In relation to this, the redirection of glucose to the pentose phosphate pathway increases the availability of NADPH that functions as a cofactor for antioxidant enzymes, and the regulation of the Kreb’s cycle modulates the concentration of α-ketoacids that in the presence of ROS are decarboxylated to produce non-toxic byproducts^11^.

The biogenic polyamines putrescine (Put) and spermidine (Spd) are ubiquitous in nature and essential for most of all living organisms^13^. Their synthesis starts with the decarboxylation of the amino acids arginine and ornithine by the enzymes arginine decarboxylase and ornithine decarboxylase, respectively, to render Put as the final product. Spd is later formed from Put by adding two aminopropyl groups through the catalytic activity of the enzyme Spd synthase. Polyamines are positively charged at physiological pH, which allows them to bind to polyanions like RNA, DNA, proteins, and lipids. As a result, they have been linked to crucial biological processes, including the regulation of transcription, translation, cell growth, and stress response. In this respect, studies on the model *Escherichia coli* demonstrated that polyamine synthesis is required to reduce the damage caused by oxidative stress^14–17^.

This view is challenged by our recent meta-analysis of transcriptome data, which demonstrates that the repression of the genes involved in the anabolism of polyamines occurs in diverse bacterial species shortly after cells are exposed to H_2_O_2_^18^. Therefore, more work is yet to be done in order to fully understand the regulatory features that govern the homeostasis of polyamines under oxidative conditions. We do have, however, some hints of the mechanisms underlying the protective effects mediated by polyamines. Thus, Spd was proposed to protect *Pseudomonas putida* against H_2_O_2_ through the stimulation of the formation of siderophores in the cytoplasm^19^, thus reducing the availability of free iron molecules and preventing H_2_O_2_ formation through the Fenton reaction. Spd also seems to mediate the protection of *P. aeruginosa* against the oxidative damage caused by bactericidal antibiotics due to its ability to reduce the binding of these molecules to bacterial membranes^20^. At last, it has also been suggested that polyamines could function directly by quenching the activity of ROS against cellular components^21,22^.

These data highlight the importance of polyamines for oxidative stress tolerance in bacteria. However, none of the studies mentioned previously were undertaken using phytopathogenic bacteria, even though the ability to overcome this kind of stress is crucial for their survival in plant tissues. In this research, we examined the functions of polyamines in the oxidative stress response of the bacterial phytopathogen *Pseudomonas syringae*. This species is a well-known and worldwide distributed phytopathogen causing diseases in a vast number of economically important plant cultivars^23^. By using the model strain *P. syringae* pv. tomato DC3000 (*Pst* DC3000), we showed that cells exposed to H_2_O_2_ modify the ratio between internal and external polyamine contents. A further examination of different mutant strains perturbed in polyamine synthesis revealed that this is linked to a higher production of catalases and the modulation of outer membrane stability. Therefore, our data are consistent with a model where polyamines protect bacterial cells from oxidative molecules by stimulating their degradation as well as preserving cell integrity.

## Results

### Pst DC3000 increases extracellular putrescine concentrations under oxidative conditions

**Figure 1:**
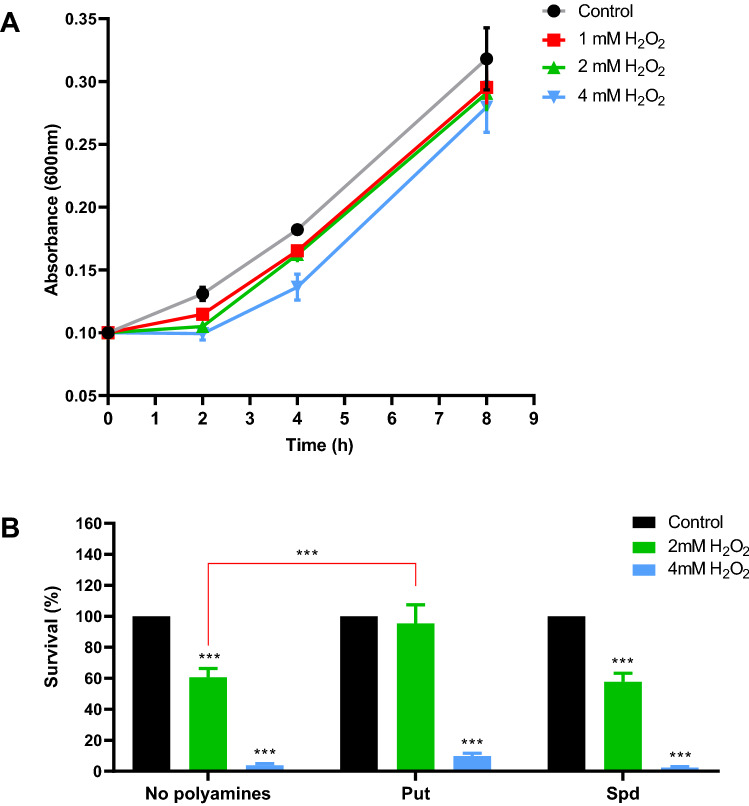
(**A**) Effect of H_2_O_2_ on bacterial growth. *Pst* DC3000 was grown in M9 under control condition or with the amendment of H_2_O_2_ at the indicated concentrations. Growth was followed by the determination of the absorbance of cultures at 600 nm. (**B**) Cell survival in the presence of H_2_O_2_. *Pst* DC3000 cultures in M9 (initial OD_600_ = 0.2) were incubated for 1 h in under control conditions or in the presence of 2 or 4 mM H_2_O_2_ either without or with Put or Spd supplementation at a final concentration of 2 mM. After this period, CFUs were determined and expressed as a percentage of the values quantified in control cultures (black bars). Statistically significant differences were evaluated by one-way ANOVA followed by Tukey´s multiple comparison tests. Asterisks on top of bars indicate differences between controls and H_2_O_2_-treated cells. Statistical differences between polyamine-treated cells are indicated with red square brackets. ***p < 0.001.

The effects of an oxidative environment on *Pst* DC3000 growth were initially investigated. Thus, the optical density (600 nm) of cultures growing in M9 minimal medium were recorded for 8 h either under control conditions or with the addition of increasing concentrations of H_2_O_2_. As shown in Fig. 1A, the concentrations of H_2_O_2_ being tested seemed to delay the onset of the exponential phase, even though growth rates eventually recovered and were indistinguishable from those observed under control conditions. We tried to discern whether the delayed lag phase is due to cell death or rather to growth arrest by assessing bacterial survival after their incubation in 2 and 4 mM H_2_O_2_ for 1 h. In this case, 2 mM H_2_O_2_ diminished bacterial survival by 40%, whereas 4 mM H_2_O_2_ caused a considerably higher reduction in cell viability when compared to the control (Fig. 1B, first set of bars). These results demonstrate that oxidative stress results in *Pst* DC3000 cell death, but they also suggest the existence of a defense system that partially protects bacterial cells and enables them to adapt to these harmful conditions.

**Figure 2:**
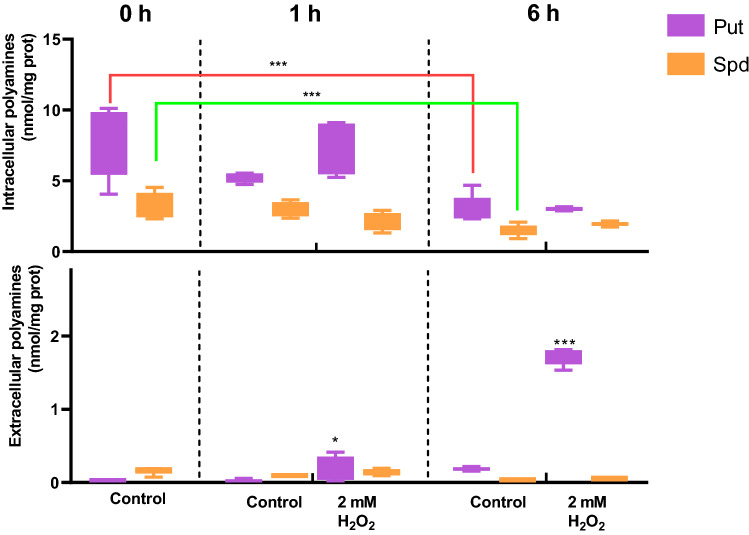
Concentrations of intracellular and extracellular polyamines in *Pst* DC3000 in response to oxidative stress. Cells were grown for 6 h in control M9 media or M9 supplemented with H_2_O_2_ at a final concentration of 2 mM. Polyamine concentrations were determined in the intracellular (top graph) and extracellular (bottom graph) spaces as described in Materials and Methods. Concentrations of polyamines were compared to those obtained under control conditions at 0 h using the Student’s t test. (*p < 0.05, **p < 0.01).

To investigate whether the adaptation to oxidative stress entails a change in the homeostasis of polyamines, the extracellular and intracellular concentrations of these compounds were quantified. Put was the main polyamine in cells growing under control conditions, and their intracellular concentrations were reduced after the first hour of culture (Fig. 2). Even though the levels of Spd were comparatively lower than those of Put throughout the period under analysis, they also followed a similar behavior. In turn, the extracellular amounts of polyamines remained unaltered. Interestingly, challenging cells with H_2_O_2_ leads to similar changes in intracellular polyamines as observed in control cells, but a remarkable accumulation of extracellular Put was observed. As a result, we speculated that Put secretion could help *Pst* DC3000 to endure oxidative environments.

To further investigate the effects of external polyamines, we evaluated bacterial survival when challenged with H_2_O_2_ for cells cultivated in media supplemented with Put or Spd (Fig. 1B). Both polyamines were used at a concentration of 2 mM, which had no impact on the rates of cell growth (Fig. S1). Interestingly, while the presence of Put in the culture media increased bacterial tolerance to H_2_O_2_, Spd had no significant effect on the susceptibility to H_2_O_2_. On the basis of previous reports, it could be argued that Put acts to shield cells by directly quenching the oxidative effects of H_2_O_2_^21^. However, our analysis using the fluorescent probe Amplex Red shows that neither Put nor Spd diminished H_2_O_2_ oxidation (Fig. S2). Therefore, other oxidative stress tolerance mechanisms than quenching could be mediated by extracellular Put, a possibility that is further investigated in the following sections.

### Perturbation of polyamine synthesis in Pst DC3000 leads to contrasting phenotypes under oxidative stress.

**Figure 3:**
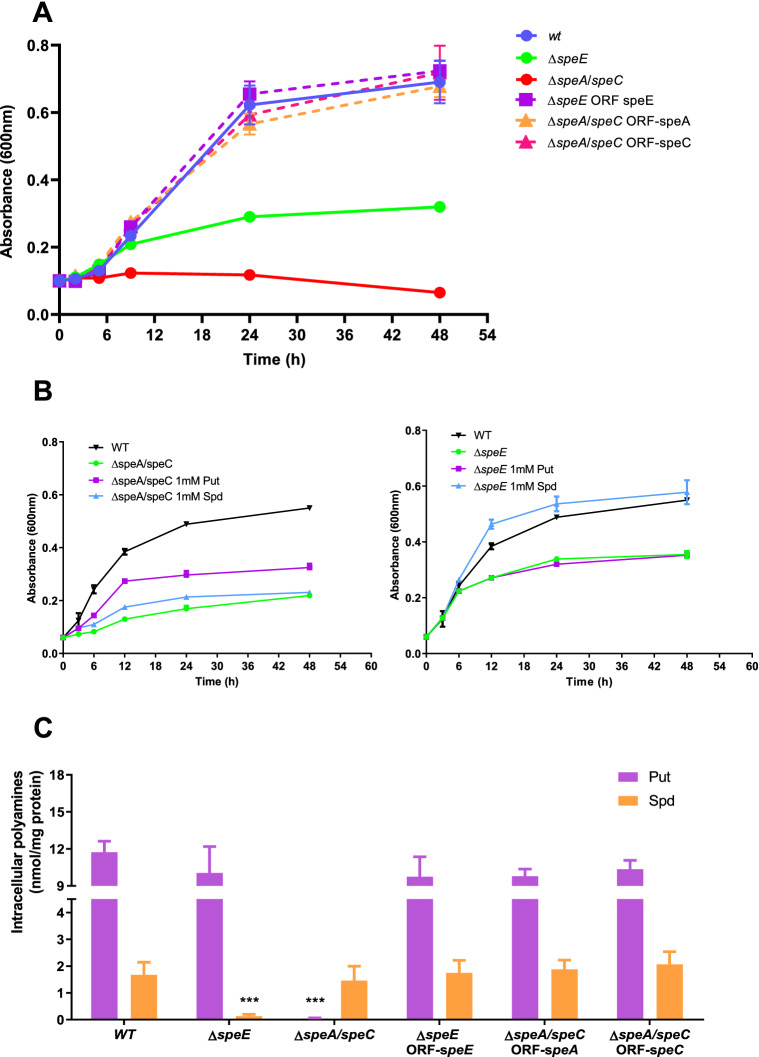
Growth (**A**, **B**) and intracellular polyamine levels (**C**) in wild type (WT), mutant strains, and reconstituted mutants of *Pst* DC3000. (**A**) Cells were grown in M9 and the optical density at 600 nm recorded for 48 h. (**B**) The same protocol as in A was used but media were amended the indicated polyamines at a final concentration of 1 mM. (**C**) Samples taken after 12 h were used to determine the intracellular levels of polyamines as described in Materials and Methods. Concentrations in mutants and reconstituted strains were compared to those observed in the wild type using the Student’s t test. ***p < 0.001.

Different mutant strains affected in the production of Put and Spd were created to gain a better understanding of the role that polyamines play in oxidative stress tolerance. In this respect, we deleted the arginine decarboxylase (*speA*) and ornithine decarboxylase (*speC*) genes in single and double mutants (∆*speA*, ∆*speC*, and ∆*speA*/∆*speC*) in an attempt to perturb the synthesis of Put and Spd, whilst Spd production was blocked by interrupting the Spd synthase gene (*speE*). In comparison to wild type cells, single interruptions of *speA* or *speC* did not cause any changes in the intracellular contents of polyamines or bacterial growth rate under control conditions (Fig. 3A,C). On these grounds, we propose that polyamine requirements are met by the decarboxylation of arginine or ornithine when either of these alternative routes is disrupted.

Deleting both genes simultaneously in the ∆*speA*/*speC* strain resulted in a significant reduction in cell growth and very low levels of intracellular Put, but counterintuitively, we found that the Spd levels were comparable to those in the wild type despite the fact that this strain should not be able to synthesise Spd because the lack of the substrate’s reaction. To explain this observation, it should be considered that free Spd could have different sources. For instance, it might be residual Spd absorbed during the preparation of the inocula in LB, which contains polyamines in its composition^24^. An alternative explanation consists in the release of free Spd from the fraction that exists conjugated to organic compounds^25,26^. Finally, we must include the possibility that *P. syringae* possesses a novel Spd biosynthesis pathway as were previously identified in *V. cholerae* and *some Pseudomonads*^19,27,28^*.*

The supplementation of the culture media with Put, in contrast to the addition of Spd, partially restored the growth of the double mutant (Fig. 3B). However, complementation of this double mutant with *speA* or *speC* completely restored intracellular polyamine concentrations and conferred growth rates identical to the wild type, in support of the idea that any of the pathways producing Put is sufficient to maintain growth (Figs. 3A,C). In turn, loss of *speE* reduced the intracellular contents of Spd, although Put levels remained unaffected. This strain shows a lower growth rate compared to the wild type, which was totally restored with the addition of Spd (but not Put) to the culture media or by complementation with *speE* (Fig. 3B,C). Considering these findings collectively, we drew the conclusion that even though it appears that Put plays major roles during bacterial growth, Spd is still needed to meet cell requirements. In other words, each polyamine could perform different functions in this process that cannot be totally substituted by the other.

**Figure 4:**
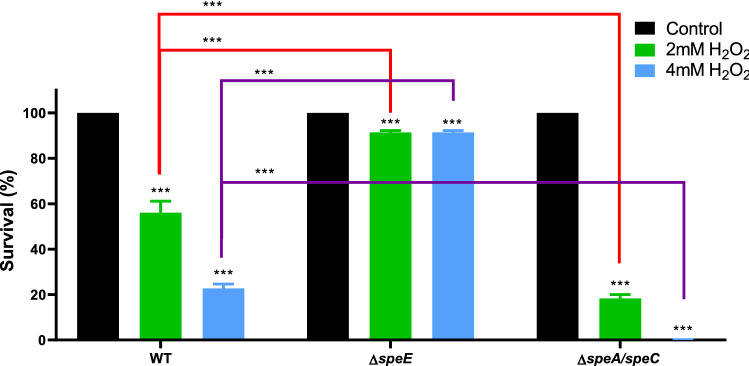
Cell survival in the presence of H_2_O_2_. Cultures of *Pst* DC3000 and derivative mutant strains in M9 (initial OD_600_ = 0.2) were incubated for 1 h under control conditions or in the presence of 2 or 4 mM H_2_O_2_. After this period, CFUs were determined and expressed as a percentage of the values quantified in control cultures (black bars). Statistically significant differences were evaluated by one-way ANOVA followed by Tukey’s multiple comparison tests. Asterisks on top of bars indicate differences between controls and H_2_O_2_-treated cells. Statistical differences between strains are indicated with red and purple square brackets. ***p < 0.001.

**Figure 5:**
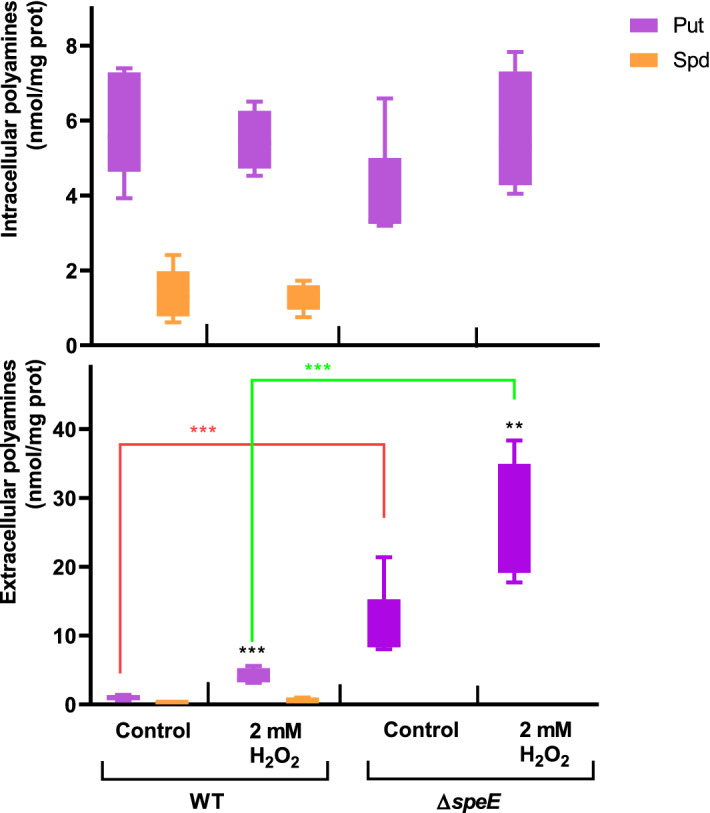
Effect of H_2_O_2_ in the intracellular and extracellular polyamine concentrations of the wild type and ∆s*peE* strain. Cells were grown for 6 h in control M9 media or M9 supplemented with H_2_O_2_ at a final concentration of 2 mM. Polyamine concentration was determined in the intracellular (top graph) and extracellular (bottom graph) compartments as described in Materials and Methods. Concentrations of polyamines in treated cells were compared to those obtained under control conditions using the Student’s t test. In addition, statistically significant differences in concentrations between strains under the same treatment are shown in red and green square brackets (**p < 0.05, ***p < 0.01).

The ∆*speA* and ∆*speC* mutants show the same phenotype in response to oxidative stress as the wild type (Fig. S3), but the deletion of these genes in the ∆*speA*/*speC* strain confers high susceptibility (Fig. 4). Complementation of genes in the mutant strains restored the wild type phenotype (Fig. S4B). Strikingly, the ∆*speE* mutant showed a tolerant phenotype and complementation of this strain with a plasmid expressing *speE* reestablished susceptibility (Fig. 4 and S4A,B). We assessed the levels of Put in this strain in response to H_2_O_2_ to determine whether a difference in the concentrations of the polyamine may explain its tolerance. Similar to the alterations observed in the wild type, the intracellular fractions of Put are maintained in ∆*speE* under oxidative conditions while its extracellular concentrations increased (Fig. 5). However, the extracellular contents of Put were higher in the mutant strain both under control conditions as well as in the presence of H_2_O_2_. These results indicate that the reduction in the synthesis of Spd and/or the accumulation of Put at the extracellular space play a part of the oxidative stress defense.

### Outer membrane stability is enhanced in the ∆*speE* mutant

Membrane permeability and integrity are closely related^10,29^. In this trend, it has been demonstrated that polyamines can substitute for membrane-bound cations such as Ca^+2^ and Mg^+2^, improving membrane stability and changing its permeability as well as protecting membranes from the impact of oxidizing agents^20,30,31^. Based on this data, we hypothesized that increased extracellular Put contents could be responsible for the improved stress tolerance of the ∆*speE* mutant. To corroborate this concept, we first compared the levels of polyamines attached to the outer membrane of this strain to those of the wild type, and also evaluated outer membrane integrity as described by Yang et al.^32^ in which the optical density of cultures is assessed after the addition of a membrane-disrupting solution of SDS.

**Figure 6:**
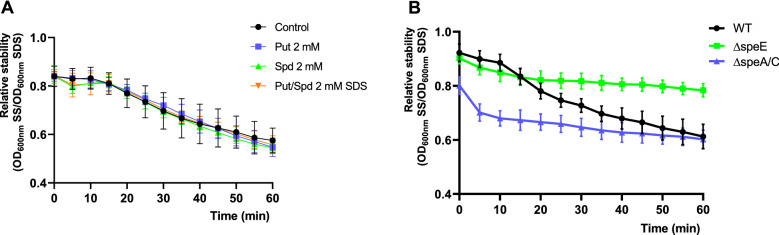
Effects of polyamines on the stability of the outer membrane. (**A**) Cells were incubated in Put or Spd alone or in combination for 1 h before incubation in saline solution (SS) or 0.1% SDS (SDS), and the OD_600_ of the culture recorded during 60 min. Membrane stability is expressed as the absorbance in SDS relative to that in SS. (**B**) wild type and mutant strains were analyzed following the same approach as in (**A**).

Our analysis shows that the quantities of Put bound to the outer membrane of ∆*speE* are similar to those seen in the wild type (Fig. S5). In addition to that, incubation of wild type cells in the presence of Put or Spd, alone or combined, did not change membrane stability (Fig. 6A). The same results were obtained by using higher polyamine concentrations (up to 5 mM) or washing the membrane-bound polyamines with 1 M NaCl before the incubation in polyamines (Fig. S6). Thus, it is plausible that the accumulation of Put at the extracellular surface plays a minor role in stress tolerance. Nevertheless, further investigation demonstrated that the ∆*speE* mutant is more stable in the presence of SDS (Fig. 6B), whereas the ∆*speA*/∆*speC* double mutant has a very low membrane stability (most of the cells were disrupted immediately after the addition of SDS). Therefore, we conclude that membrane integrity may explain, at least partially, the enhancement of stress tolerance in ∆*speE*. Whether this is a consequence of their reduced Spd content should be evaluated.

### Spermidine synthesis disruption induces catalase activity

**Figure 7:**
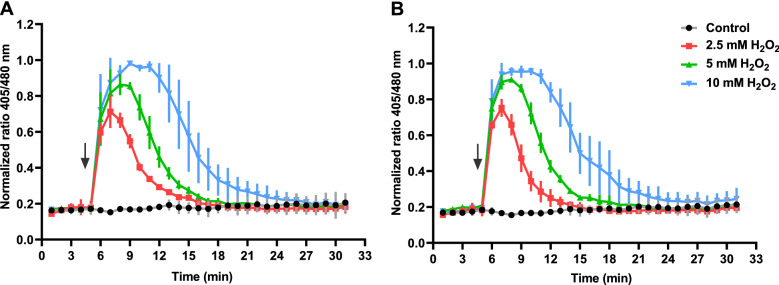
Kinetic analysis of the intracellular redox in Pst DC3000 state in response to H_2_O_2_. Cells of the wild type (**A**) and ∆*speE* (**B**) strains expressing roGFP were exposed to different concentrations of H_2_O_2_ (arrows indicate the time of addition). Data points represent the intracellular redox state as the quotient of the excitation fluorescence values at 405 and 480 nm.

Cells can avoid the cytotoxicity caused by ROS by producing antioxidative compounds and degradative enzymes^6^. The tripeptide glutathione is the major antioxidant molecule in bacterial cells, and it is crucial to maintain the intracellular redox state^33^. Therefore, if the induction of a glutathione-dependent mechanism underlies the ∆*speE* mutant's increased tolerance to H_2_O_2_, its intracellular redox state should recover to normal levels more quickly than the wild type. To test this possibility, we explored the variations in the cytoplasmic redox potential in *Pst* DC3000 by constitutively expressing the redox-sensitive variant roGFP, which changes its excitation peaks in response to the oxidation rate of two specific cysteine residues^34^. Importantly, the oxidation of these residues depends on the glutathione potential. Our experiments demonstrate that both the wild type and the ∆*speE* mutant were equally capable of restoring the intracellular redox equilibrium at the same rate (Fig. 7). We thus disregarded the contribution of glutathione to stress tolerance.

**Figure 8:**
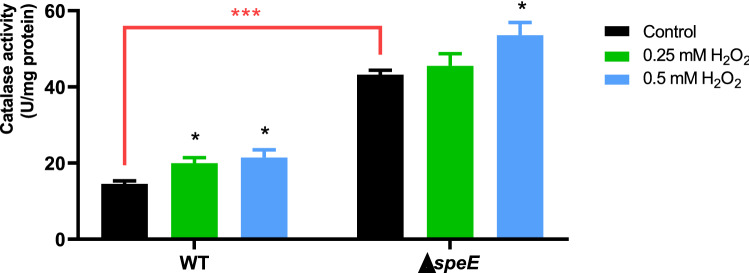
Catalase activity in *Pst* DC3000 in response to H_2_O_2_. Cells from the wild type and ∆*speE* mutant strain grown in M9 were exposed to different concentrations of H_2_O_2_ for 1 h. Catalase activity was determined in cell extracts through the decomposition of H_2_O_2_ by following the decrease in absorbance at 240 nm. Data from each strain growing under oxidative conditions were compared to their respective control (without H_2_O_2_-amendment) using the Student’s t test. Additionally, controls from different strains were compared (red square bracket). *p < 0.05, ***p < 0.001.

Catalases, which catalyze the breakdown of H_2_O_2_ to water, are some of the main enzymes protecting bacteria against H_2_O_2_^6,35^. In order to determine if these enzymes are regulated by polyamines, we evaluated the catalase activity in the wild type and the ∆*speE* mutant strains. As shown in Fig. 8, even though both strains respond to H_2_O_2_ with the induction of catalase activity, the ∆*speE* mutant exhibits remarkably higher baseline levels. These results show that the reduction in the synthesis of Spd results in the activation of catalase activities.

**Figure 9:**
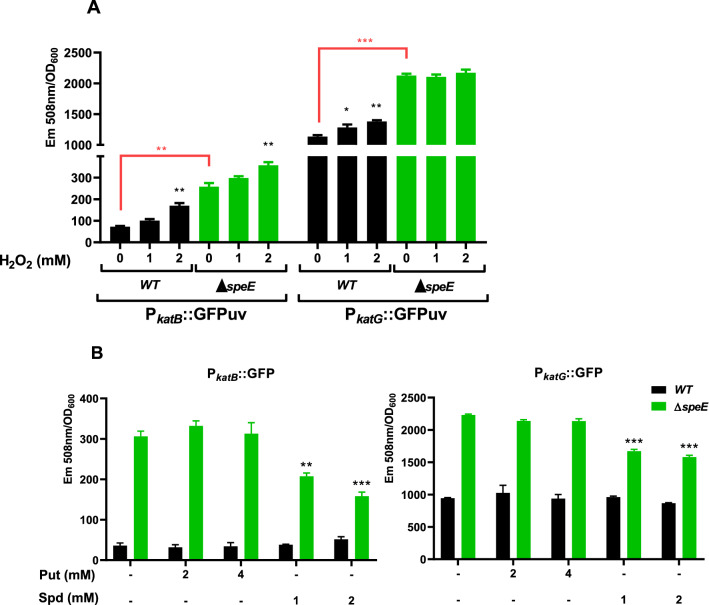
Catalase promoter activities in *Pst* DC3000 in response to H_2_O_2_ and polyamines. (**A**) Cells from the wild type and ∆*speE* mutant expressing the mentioned reporter constructions were exposed to different concentrations of H_2_O_2_ and the emission of fluorescence at 508 nm determined after 1 h. The same approach was used in (**B**), but cells were challenged with 2 mM of H_2_O_2_ in the absence or presence of polyamines at the indicated concentrations. Data of fluorescence in H_2_O_2_-treated cells were compared to their respective controls (without H_2_O_2_ or polyamine-amendment). Additionally, values in control cells from different strains were compared in (**A**) (black square brackets). **p < 0.01, ***p < 0.001.

Three catalases have been identified in *Pst* DC3000, being the products of *katG* and *katB* the major enzymes required to cope with oxidative stress^35^. To assess whether these isoenzymes respond to polyamines, we used reporter constructs expressing the highly fluorescent variant GFPuv^36^ under the control of the promoters of either *katG* or *katB*. Our investigations revealed that both *katB* and *katG* promoter activities are induced in the wild type strain in response to H_2_O_2_, supporting the notion that these enzymes participate in the detoxification of this compound (Fig. 9A, black bars). Interestingly, the fluorescence derived from the *katB*:*GFPuv* or *katG*:*GFPuv* constructs were noticeably higher in the ∆*speE* mutant when compared to the wild type under all conditions. Additionally, the incubation of the mutant cells in H_2_O_2_ did not promote a further induction of *katG* promoter activity, even though *katB* is further induced. These results indicate that both enzymes are highly expressed when the intracellular contents of Spd are reduced.

We next evaluated the effect of polyamine amendment on the fluorescence emission of these reporter constructions as a means to evidence a regulatory mechanism operating on the expression of catalases. This experiment shows that *katB:GFPuv* and *katG:GFPuv*-derived fluorescence were reduced in the ∆*speE* mutant in the presence of Spd, whereas Put had no effect on the reporter´s activities (Fig. 9B). In addition, adding polyamines to the wild type strain did not change the emitted fluorescence. These results suggests that Spd negatively affects the expression of catalases.

## Discussion

A large body of research demonstrates close connections between polyamines and a variety of processes controlling how cells react to both internal and external stimuli^37^. In bacteria, current knowledge indicates that both the intracellular as well as the extracellular fraction of polyamines contribute to the activation of such responses, even though the functionality of each of these fractions may be different. Thus, research on *E. coli* has demonstrated that intracellular polyamines increase general protein synthesis by facilitating the assembly of the 30S ribosomal subunits, promote at the level of transcription the expression of different proteins implicated in bacterial growth and fitness, and contribute to adjustments of the cytoplasmic pH^13,38,39^. In turn, extracellular polyamines are involved in processes such as cell-to-cell communication in *Dickeya zeae*^40^ and stabilization of cell membranes in several bacterial species^20,30,41–43^. Therefore, precise modulation of the intra- and extracellular concentrations of polyamines is required for bacteria to adapt to the wide variety of conditions they encounter throughout their lifespan. In view of these facts, our investigation intended to clarify the function of polyamine homeostasis in the response to oxidative stress.

We used the phytopathogenic bacteria *Pst* DC3000 as a model, which initially colonizes and thrives as a saprophyte on the surface of plant organs, and eventually enters plant tissues through wounds or natural openings like stomata^44^. Plants identify the pathogen´s presence during this process and coordinate the activation of defense systems, which ultimately expose invading cells to oxidative stress in an effort to stop their proliferation and the spread of the disease. This defensive response results from the activation of plant enzymes that build up ROS around the bacterial cells, as well as from the activity of antimicrobial plant metabolites that alter the bacterial redox state and raise the internal concentration of ROS^45^.

We first tested *Pst* DC3000's resistance to H_2_O_2_ using in vitro cultures in M9 minimal medium. The concentrations of H_2_O_2_ used in this experiment delayed the onset of the bacterial exponential growth phase as a result of a reduction in cell survival, but had no discernible impact on growth after that point. This shows that *Pst* DC3000 is able to deploy detoxifying mechanisms to avoid the negative effects provoked by H_2_O_2_. In fact, it shares a similar tolerance to H_2_O_2_ as *E. coli* and *P. aeruginosa*^46,47^, which can withstand relatively higher concentrations of this compound in comparison to other bacterial species such as *Salmonella enterica*^48^ and *P. putida*^49^.

Put and Spd were the main polyamines found in *Pst* DC3000 cells growing in M9. Their inner concentrations were reduced after 6 h of culture, whereas small amounts were detected at culture supernatants. Interestingly, even though the intracellular amounts of polyamines are not modified in response to H_2_O_2_, a remarkable increment in extracellular Put was observed. On these grounds, we envisioned that the homeostasis of polyamines is required to cope with the detrimental effects caused by oxidative stress conditions. To test this idea, we assessed the phenotype of strains perturbed in the synthesis of Put (∆*speA*, ∆*speC*, and ∆*speA/C*) and Spd (∆*speE*). Deletion of *speA* or *speC* had no impact on cell proliferation or polyamine levels, proving that any of these routes can meet polyamine requirements. However, simultaneous deletion of both genes resulted in a significant decrease in Put concentration and cell growth rate. The growth of the ∆*speA/C* strain is completely restored by the expression or either *speA* or *speC*, and partially with the supplementation of the culture media with Put. Conversely, the absence of *speE* led to a noticeably lower contents of Spd while causing a less severe decline in growth. In this case, growth reach the levels of the wild type strain by adding Spd to the media or expressing *speE*. Therefore, it appears that although both Put and Spd are required to maintain bacterial development, each polyamine might perform crucial functions that cannot be substituted.

Unexpectedly, the *∆speE* strain demonstrated higher tolerance to H_2_O_2_, which contrasts with the susceptibility exhibited by the double mutant ∆*speA/C*. Additional examination of the changes in polyamine contents in response to oxidative stress indicated that the intracellular contents of Put are not modified in the *ΔspeE* strain as is also observed in the wild type, but relatively higher levels of this polyamine are found under normal and oxidative environments at the extracellular space. This suggests that reducing the intracellular Spd fraction and/or raising the amounts of external Put are involved in the antioxidative response. Recent research by Kumar et al.^50^ has shown that free Spd may directly oxidize intracellular iron in *E. coli* to produce superoxide radicals, which explains the toxicity caused by high concentrations of this polyamine. The possibility that the reduction in the expression of *speE* and the secretion of its substrate (Put) is intended to avoid superoxide radical imbalance in *P. syringae* was not assessed in this work, but it deserves future research.

The outer membrane of Gram negative bacteria is asymmetric, where the inner leaflet (facing the periplasm) is composed by phospholipids and the outer layer is formed by glycolipids, including anionic lipopolysaccharides (LPS)^51^. The enhancement of its stability and impermeability is important to endure stress conditions. For instance, bacteria such as *Salmonella typhimurium* modulate outer membrane permeability to overcome the oxidative conditions imposed by H_2_O_2_^43,51^. This has led researchers to propose the targeting of membrane integrity as a therapeutic approach^9,52^. Importantly, Johnson et al.^20^ demonstrated that Put and Spd bind to the outer membrane of *P. aeruginosa*, and that Spd replaces divalent cations to protect the membrane against antimicrobial peptides and lipid peroxidation.

To discern whether the external fractions of polyamines fulfill similar roles in *Pst* DC3000, we examined membrane stability in mutant strains and the effects of polyamine amendment to the culture media. Interestingly, the ∆*speE* mutant shows enhanced outer membrane stability when compared to the wild type strain, which cannot be explained on the basis of an increment in the levels of Put attached to membranes or a higher accumulation of this polyamine at the exterior of cells. This is because identical levels of membrane-bound polyamines were found between ∆*speE* and the wild type, and besides, the addition of Put or Spd to the culture media did not modify membrane stability even when using cells previously washed with NaCl 1 M to detached membrane-bound polyamines. Therefore, we concluded that the effect provoked by deleting the s*peE* gene on membrane integrity did not depend on the higher contents of Put found extracellularly but is a consequence of reduced intracellular levels of Spd.

We also showed that polyamines don´t have an antioxidative quenching activity against H_2_O_2_, suggesting that external Put levels plays a minor role in direct defense against oxidative stress. This conclusion seems to contradict our finding that Put supplementation favors cell survival in the presence of H_2_O_2_. However, it should be considered the possibility that the effects observed by the addition of polyamines to the culture media are not due to their extracellular functions but rather to their likely incorporation and potential effects on cell physiology. This hypothesis should be evaluated by the use of strains affected in polyamine transport.

Another important mechanism associated with oxidative stress tolerance is the ability to metabolize or reduce ROS toxicity. This involves the production of thiol-containing substances like glutathione and antioxidant enzymes that usually work cooperatively to maintain the redox balance inside the cell^6,53^. Importantly, polyamines stimulate in *E. coli* the induction of the genes *gshA* and *katG*, which are key enzymes involved in glutathione anabolism and H_2_O_2_ detoxification, respectively^54,55^. Because of this, we hypothesized that deletion of *speE* might also entail the induction of any of these components of the antioxidant system. Our experiments ruled-out the participation of glutathione in the antioxidant system. This is based on the observation that the wild type and ∆*speE* strains are equally able to mitigate the effects of H_2_O_2_ on the internal redox status, as measured using a reporter construction expressing the roGFP fluorescent protein. In turn, further research demonstrated that higher basal levels of catalase activity occur in the ∆*speE* strain compared to the wild type, suggesting that stress tolerance is related to higher rates of H_2_O_2_ detoxification.

Three catalase genes are found in the genome of *Pst* DC3000, but only the products of *katB* and *katG* are crucial for H_2_O_2_ breakdown^35^. Whereas *katG* is likely found at the cytoplasm, *katB* functions in the cytoplasm and the periplasmic space. According to our investigation of *katB* and *katG* promoter activity, the predominant housekeeping enzyme in this strain is KatG and both genes are strongly activated in the ∆*speE* strain. Additionally, only *katB* promoter activity demonstrated H_2_O_2_-dependency in ∆*speE*, whereas H_2_O_2_ increased the *katB* and *katG* promoter activities in the wild type. It is interesting to note that whereas Put or Spd have no impact on the promoter activity of *katB* or *katG* in the wild type strain, their activity is decreased in the ∆*speE* mutant strain when Spd (but not Put) is added. Therefore, the existence of a regulatory mechanism where Spd functions as a negative modulator of catalase activity could explain the reason behind the stress tolerant phenotype observed in the ∆*speE* strain. In this trend, our previous co-expression analyses on several *P. syringae* strains demonstrated a negative correlation between polyamine biosynthetic genes and catalases^18^. Besides, it was demonstrated that *speE* is downregulated in *P. syringae* B728A and *P. aeruginosa* PAO1, as well as in non-related species such as *E. coli* and *Salmonella enterica* in response to H_2_O_2_^48,56–58^. This observation implies the existence of a wide distributed regulatory mechanisms linking Spd synthesis reduction and catalase induction.

**Figure 10:**
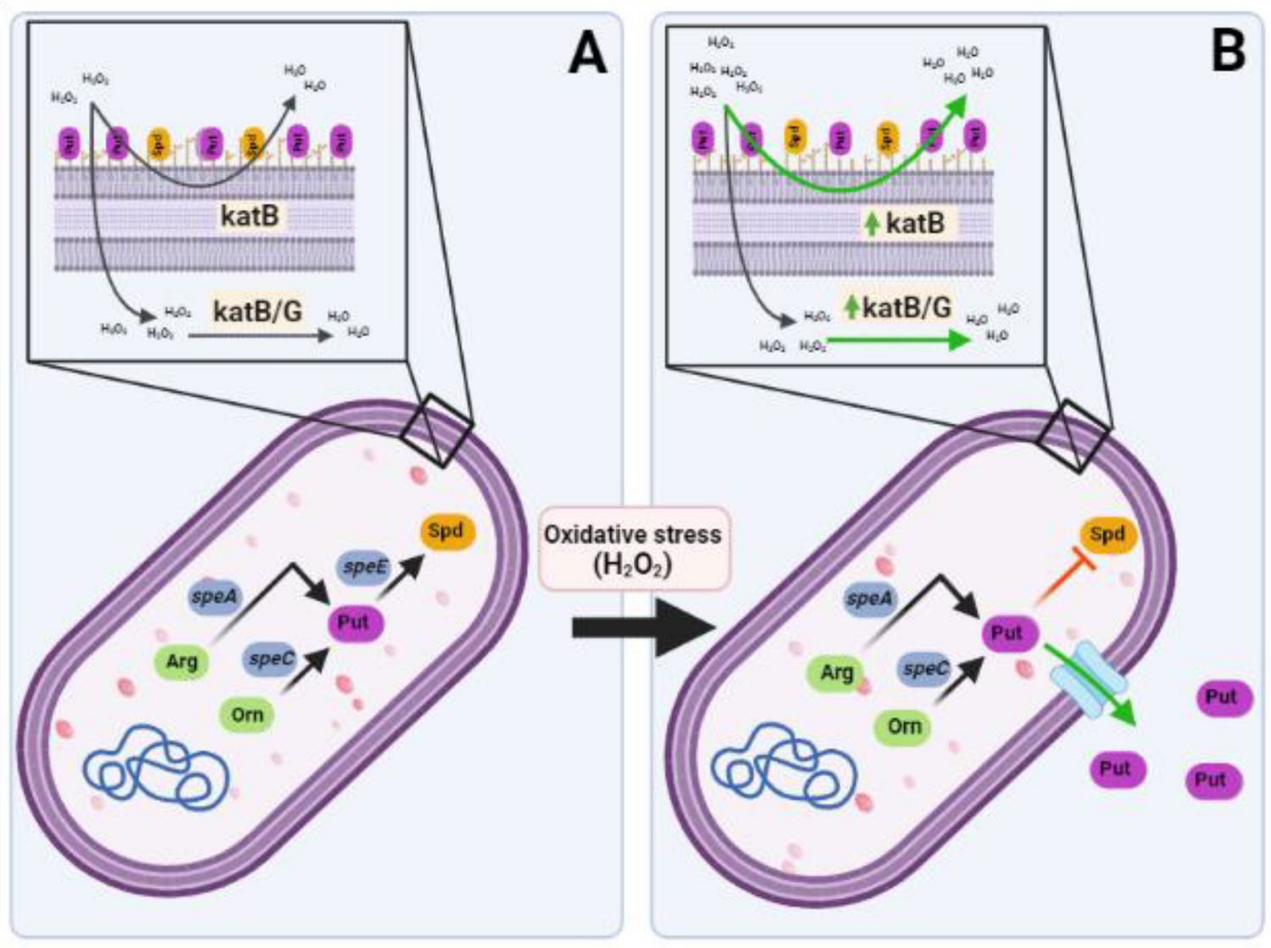
Diagram illustrating the alterations in polyamine metabolism and their relationships to the antioxidant response in *Pst* DC3000. (**A**) During optimal growth and development, bacteria usually deal with small amounts of extracellular concentration of H_2_O_2_, but also with autogenously generated intracellular amounts of this reactive species. Under these conditions, external H_2_O_2_ (that may permeate through membranes) and internal H_2_O_2_ are quickly metabolized by catalases. (**B**) During plant invasion, bacteria is recognized by the plant surveying system and a series of defense mechanisms are deployed imposing oxidative stress to the microbe. This implies the activation of plant enzymes that rise the extracellular concentrations of H_2_O_2_ and the production of antimicrobial compounds that provoke an increment in the internal amounts of this compound. In response to these highly oxidative conditions, bacteria secretes Put to the extracellular medium. Interrupting the synthesis of Spd could also contribute to this response, as this leads to the induction of catalase activity. Besides, the outer membrane stabilizes as a result of this process, which may affect its permeability and reduce H_2_O_2_ entry.

Altogether, we have shown that exposure to oxidative stress results in an increase in the concentration of extracellular Put in *P. syringae*. Besides, we demonstrated that perturbing the synthesis of Spd promotes oxidative stress tolerance, which correlates with our previous report describing the downregulation of Spd synthesis as a response to H_2_O_2_ in several bacterial species^18^. This is associated to the stabilization of the outer membrane and the induction of catalases (Fig. 10). Thus, we conclude that these changes in the homeostasis of polyamines may participate in a mechanism controlling the redox balance of the cell and ensure bacterial viability under oxidative conditions. However, it should be considered that the model depicted here could differ in the plant environment since polyamines, whether derived from the bacterial or plant metabolism, may have dissimilar functions. For instance, it has been shown that Put accumulates in plant tissues during bacterial infections^21,24,59^. This effect, for some bacterial species, is a consequence of the action of secreted effectors that increments plant arginine decarboxylase activities to promote Put production and the activation of defense responses, which ultimately reduce bacterial cell proliferation^59,60^. In connection with this, it has been discovered that the generation of the toxin phevamine A by *P. syringae* inhibits the potentiation of ROS production that is mediated by polyamines in Arabidopsis^61^. These findings collectively imply that bacteria alter their own polyamine metabolism to protect themselves from oxidative stress, but these changes may also have adverse impacts by strengthening plant defensive mechanisms. The goal of our next research is to understand how bacteria manage the delicate balance between the beneficial and harmful roles played by polyamines during plant invasion.

## Methods

### Bacterial strains and plasmids

Table 1 lists bacterial strains and plasmids included in this study. Bacteria were routinely grown in LB or minimal medium M9^62^ at 28 °C, supplemented, when necessary, with rifampicin (50 µg/mL), kanamycin (50 µg/mL), or gentamycin (40 µg/mL in agar plates and 30 µg/mL in liquid medium).

**Table 1:** Bacterial strains and plasmid used in this study.

Strains or plasmids	Relevant characteristic	Source or reference
Strains		
*Pst* DC3000	*Pseudomonas syryngae* pv. tomato DC3000, Rif^R^	Buell et al.^70^
∆s*peA*	*Pst* DC3000 unmarked mutant lacking s*peA* gene, Rif^R^	This work
∆s*peC*	*Pst* DC3000 unmarked mutant lacking s*peC* gene, Rif^R^	This work
∆s*peE*	*Pst* DC3000 unmarked mutant lacking s*peE* gene, Rif^R^	This work
∆s*peA/speC*	*Pst* DC3000 unmarked mutant lacking s*peA and speC* genes, Rif^R^	This work
One Shot® OmniMAX™ 2 T1R	*Escherichia coli* F′^28^*mcr*A Δ(*mrr hsd*RMS-*mcr*BC) Φ 80(*lac*Z)ΔM15 Δ(*lac*ZYA-*arg*F)U169 *end*A1 *rec*A1 *sup*E44 *thi-*1 *gyr*A96 *rel*A1 *ton*A *pan*D	Inivtrogen ®
S17 ʎ*pir*	*Escherichia coli* Tp^R^ Sm^R^ *recA thi pro hsd* r^-^ m^+^ RP4::2-Tc::Mu::Km Tn*7 lpir*	Simon et al.^71^
Plasmids		
pFVP 25.1-roGFP	Derivative of pfpv25 expressing roGFP2, Gm^R^	van der Heijden and Finlay^69^
pK18MobsacB	Small mobilizable vector derivative of pK18, sucrose-sensitive (sacB), Km^R^	Schafer et al.^72^
pBBR1MCS-5	Derivative of the broad-host-range cloning vector pBBR1MCS; Gm^R^	Kovach et al.^73^
pORF-s*peA*	pBBR1MCS-5 derivative carrying the *speA* gene with its native promoter, Gm^R^	This work
pORF-s*peC*	pBBR1MCS-5 derivative carrying the *speC* gene with its native promoter, Gm^R^	This work
pORF-s*peE*	pBBR1MCS-5 derivative carrying the *speE* gene with its native promoter, Gm^R^	This work
*pkatG*::*GFPuv*	pBBR1MCS-5 derivative carrying *GFPuv* fused with *katG´s* promoter, Gm^R^	This work
*pkatB*::*GFPuv*	pBBR1MCS-5 derivative carrying *GFPuv* fused with *katB´s* promoter, Gm^R^	This work
pDSK-GFPuv	Derivative of pDSK-519 expressing GFPuv, Km^R^	Wang et al.^67^
pDSK-roGFP	pDSK derivative carrying the roGFP gene, Km^R^	This work

### Construction of mutant strains in *Pst* DC3000

The in-frame deletions of the genes coding for arginine decarboxylase (*speA*, PSPTO_4842), ornithine decarboxylase (*speC*, PSPTO_4572) and spermidine synthase (*speE*, PSPTO_2055) in *Pst* DC3000 were constructed following the homologous double-crossover method described by Kvitko and Collmer^63^ using the pK18mobsacB suicide plasmid. Briefly, the upstream (fragment A, ~ 1.000 bp) and downstream (fragment B, ~ 500 bp) sequences of each gene were PCR amplified with HiFi TransTaq® polymerase (TRANS) using AF-AR and BF-BR pair of primers (Table S1). Next, both fragments were PCR fused using primers AF and BR to generate a ~ 1.500 bp fragment that was inserted into linearized pK18mobsacB to generate the corresponding deletion cassette and transformed into *E. coli* OmniMAX™ (Invitrogen) competent cells by the heat shock method^64^. Primers M13-F and pK18M13-R were used to amplify fragments that were purified and sequenced to check for PCR errors and correct fusion. Plasmid transfer from *E. coli* to wild type *Pst* DC3000 was done by biparental mating using *E. coli* S17.1 harboring pK18mobsacB derivatives by the standard method^63^. Contra-selection was made in LB plates containing 5% sucrose. Single colonies were then picked and cultured in plates with either the combination of kanamycin and rifampicin or rifampicin alone to check loss of kanamycin resistance. Deletion strains were confirmed by PCR using the SeqA/SeqB pair of primers. To construct the double mutant ∆*speA*-s*peC*, deletion of *speC* was performed on the ∆*speA* single mutant.

### Polyamine isolation and quantification

Bacterial cultures were centrifuged at 10,000×*g* for 5 min and 60 μL of the supernatant were taken as a sample of the extracellular polyamine fraction. Bacterial pellets were washed two times with PBS buffer and finally resuspended in 300 μL of the same solution. 50 mg of glass beads (0.1 mm) were added, and cells disrupted by vigorous shaking for 45 s using a Cell disruptor Genie (Scientific Industries, Inc, Bohemia, NY, USA). After that, samples were centrifuged at 10,000×*g* for 5 min and 60 μL of the supernatant were collected to determine the intracellular polyamine fraction and protein quantification. Membrane-attached polyamines were isolated according to Johnson et al.^20^. Polyamine quantification was performed by derivatization with dansyl chloride as previously described by Vilas et al.^24^. Protein concentrations were determined by the Bradford assay^65^.

### Assessment of tolerance to H_2_O_2_

Overnight cultures of bacterial strains in LB were used to inoculate 5 mL of M9 medium at a final optical density of 0.1 (OD_600_). Bacteria were grown for 18 h without or with the addition of 2 mM of Put or Spd and then collected by centrifugation at 10.000×*g* by 5 min, washed in PBS buffer and resuspended in the same solution at OD_600_ = 0.12. Cell aliquots of 200 μL were loaded in 1.5 mL centrifuge tubes and subsequently 50 μL of PBS containing 0 mM, 10 mM or 20 mM of H_2_O_2_ were added to reach the final concentrations of 0 mM, 2 mM and 4 mM, respectively. Cells were incubated statically at 28 °C for 1 h. After this period, bacteria were washed with PBS and 5 mL of tenfold serial dilutions spotted in LB-rifampicin agar plates. Colony forming units were determined after 48 h and survival percentages (%) were determined as the number of UFC/ml of treated cell versus controls (PBS without H_2_O_2_ addition).

The H_2_O_2_ inhibition zone assay was performed as described by Guo et al.^35^. With this purpose, bacterial strains were grown overnight at 28 °C on M9. The cultures were washed and resuspended in fresh M9 medium, adjusted to an OD_600_ = 0.3, and mixed with 0.4% agar at 50 °C at a ratio of 1:10 (vol/vol). 5 mL of the mixture were quickly layered on plates containing 20 mL of M9 agar medium. A paper disk of 0.5 cm in diameter was then placed in the center of each plate, and 3 µL of 10% H_2_O_2_ (vol/vol) were applied to the disks. Plates were incubated at 28 °C for 48 h. The diameters of the inhibition zones were measured using ImageJ (https://imagej.nih.gov/ij/).

### Measurement of H_2_O_2_ quenching activity

The potential H_2_O_2_ quenching activity mediated by polyamines was assessed using the Amplex® Red Hydrogen Peroxide/Peroxidase Assay Kit (Invitrogen™). Thus, wells from a 96-well microplate were dispensed with 50 mL of 1 × reaction buffer/HRP working solution containing 10 mM of H_2_O_2_. Then, 50 mL of PBS buffer amended with 5, 50 or 200 mM of the specified polyamines were added to different wells and plates incubated for 30 min. Fluorescence emission was determined at 590 nm using a microplate reader and the concentration of H_2_O_2_ estimated using a standard curve as recommended by the manufacturer.

### Catalase activity

Catalase activity assay was performed as previously described by Tondo et al.^66^ with minor modifications. *Pst* DC3000 strains were grown overnight at 28 °C in M9, washed and resuspended in fresh medium. The OD_600_ was adjusted to 1.0 and H_2_O_2_ added to 5 mL of cell suspensions to reach final concentrations of 0, 0.25 and 0.5 mM. Cells were incubated for 1 h and harvested by centrifugation at 10,000×*g* for 10 min at 4 °C, washed and resuspended in 250 μL of ice-cold PBS containing 1 mM PMSF. Glass beads (0.1 mm) were used to disrupt cells as described previously. Suspensions were clarified by centrifugation at 10,000×*g* for 10 min at 4 °C. Protein concentrations in soluble cell extracts were determined and catalase activity was monitored through the decomposition of H_2_O_2_ by following the decrease in absorbance at 240 nm. The assays were performed at 25 °C in the presence of 10 mM H_2_O_2_. An extinction coefficient of 43.6 M^−1^ cm^−1^ was used to calculate the specific activity. One unit of catalase activity was defined as the amount of activity required to decompose 1 μmol of H_2_O_2_ per minute.

### Outer membrane stability assay

Outer membrane stability assays were conducted as described by Yang et al.^32^. Thus, cultures of cells at exponential phase in M9 were adjusted to an optical density (OD_600_) of 0.3 and treated with Put and Spd at the indicated concentrations for 1 h. After treatment, samples were washed with 0.85% saline solution and divided into two equal portions of 500 mL. Then, 500 mL of a sodium dodecyl sulfate (SDS) at a final concentration of 0.1% or 0.85% saline solution were added to different fractions. Cell disruption was measured in terms of OD_600_ decay at intervals of 5 min with a spectrophotometer.

### Construction of transcriptional fusions and determination of promoter activity

Predicted promoter sequences including the start codons of *katB* and *katG* were amplified using PromKatB F-R and PromKatG F-R pair of primers (Table S1), respectively. The ATG-less GPFuv sequence was amplified using the GFPuv-F and M13-F primers from the pDSK-GFPuv plasmid^67^. Gel purified amplicons were digested with *Kpn*I and ligated using T4 ligase (TRANS™). Ligated fragments were cloned into *EcoRI/PstI* linearized pBBR1MCS-5 and transformed into *E. coli* OmniMAX competent cells. Biparental mating with *E. coli* S17 was used to transform *Pst* DC3000 strains. Transformed bacteria carrying the different versions of pBBR1MCS-5 were grown overnight at 28 °C in M9. To prevent the development of siderophores that emit fluorescence at the same wavelength as GFP, in this case the medium was supplemented with 5 mM FeCl_3_. Cells were washed and resuspended in fresh medium to an OD_600_ of 0.8. Then, 100 μL of this solution were dispensed in wells of a black 96-well microplate, and 100 μL of M9 containing 0 mM, 4 mM or 8 mM of H_2_O_2_ were added to different wells. Finally, GFPuv-derived fluorescence (excitation, 410 nm; emission, 508 nm) and OD values after 2 h of incubation were recorded using a Synergy H1 Hybrid Multi-Mode microplate reader (BioTek Instruments, Inc.). Bacteria carrying the pBBR1MCS-5 empty plasmid were used to estimate fluorescent background signals and specific fluorescence intensity was calculated using the method described by Eiamphungporn et al.^68^.

### roGFP expression in bacteria and measurement of the intracellular redox state

A variant of the pDSK-GFPuv plasmid was generated by replacing the GFPuv sequence by roGFP. Briefly, roGFP sequence was amplified with roGFP-F/R pair of primers (Table S1) using pFPV25-roGFP plasmid as template^69^ and digested with *Xba*I and *PstI*. Subsequently, pDSK-GFPuv was digested with the same pair of enzymes to remove GFPuv and gel purified. Vector and insert were ligated using T4 ligase to generate the pDSK-roGFP plasmid and transformed into *E. coli* OmniMAX competent cells as described. Finally, biparental mating with *E. coli* S17 was employed to transform *Pst* DC3000 strains. Real-time intracellular redox measurement and data analysis was performed as described by van der Heijden and Finlay^69^ using cells cultivated in M9 amended with 50 mM FeCl_3_.

### Statistics

Each experiment was conducted at least twice with similar results using no less than five biological replicates. Figures show representative experiments, and the results are presented as the means ± standard deviation. Normally distributed data were analyzed using Student’s t-test or ANOVA followed by posthoc comparisons by Bonferroni or Dunnet´s test using GraphPad Prism version 8.0.0 for Windows, GraphPad Software, San Diego, California, USA (www.graphpad.com).

## Supplementary Information

Supplementary Figure S1.

Supplementary Figure S2.

Supplementary Figure S3.

Supplementary Figure S4.

Supplementary Figure S5.

Supplementary Figure S6.

Supplementary Table S1.

## Data Availability

The data that support the findings of this study are available from the corresponding author upon reasonable request.
