# Supplementary material for: Polyamine-mediated mechanisms contribute to oxidative stress tolerance in Pseudomonas syringae
Source: Sci Rep. 2023 Mar 15;13:4279. doi: 10.1038/s41598-023-31239-x (PMC10017717; doi:10.1038/s41598-023-31239-x)
Supplement: Supplementary file 1 — Supplementary Figure S1. [file 41598_2023_31239_MOESM1_ESM.pdf]

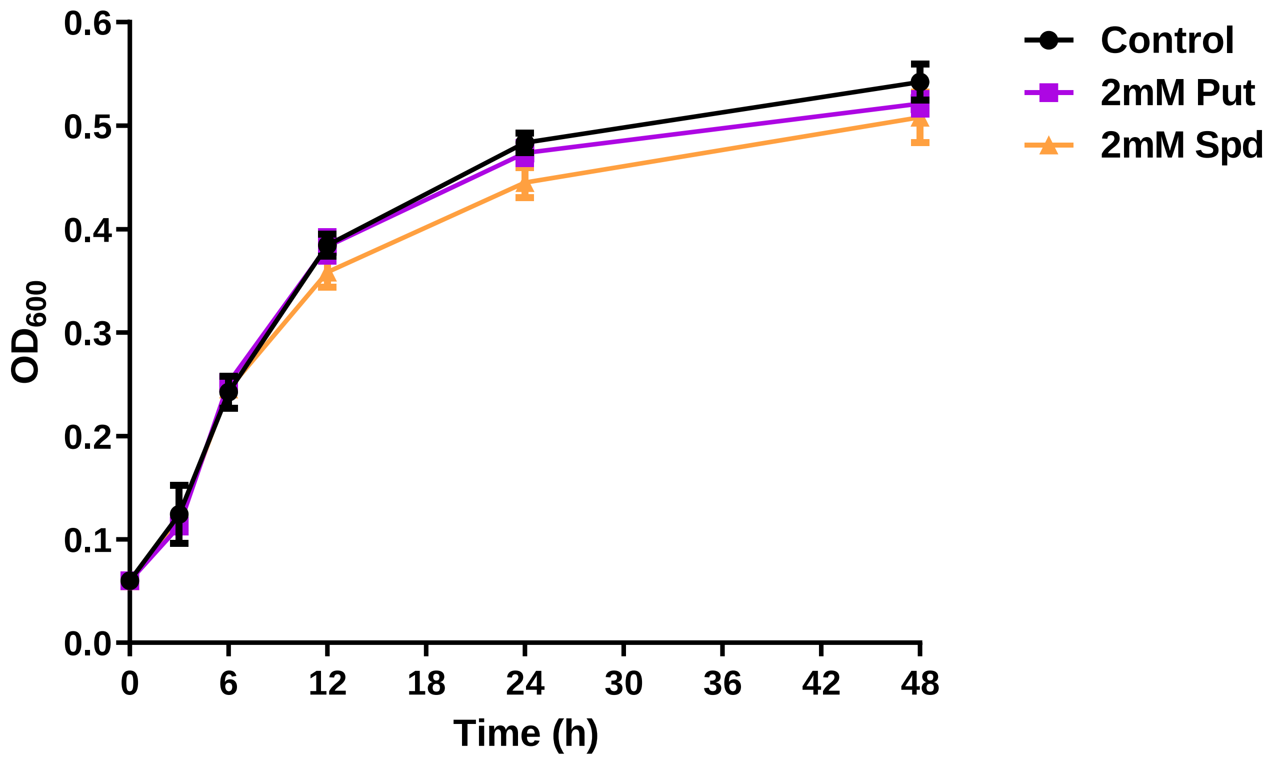

Figure S1. Effect of polyamines in bacterial growth. *Pst* DC3000 was grown in M9 under control condition or with the amendment of Put or Spd at 2 mM. Growth was followed by the determination of the absorbance of cultures at 600 nm.
