# Supplementary material for: Polyamine-mediated mechanisms contribute to oxidative stress tolerance in Pseudomonas syringae
Source: Sci Rep. 2023 Mar 15;13:4279. doi: 10.1038/s41598-023-31239-x (PMC10017717; doi:10.1038/s41598-023-31239-x)
Supplement: Supplementary file 2 — Supplementary Figure S2. [file 41598_2023_31239_MOESM2_ESM.pdf]

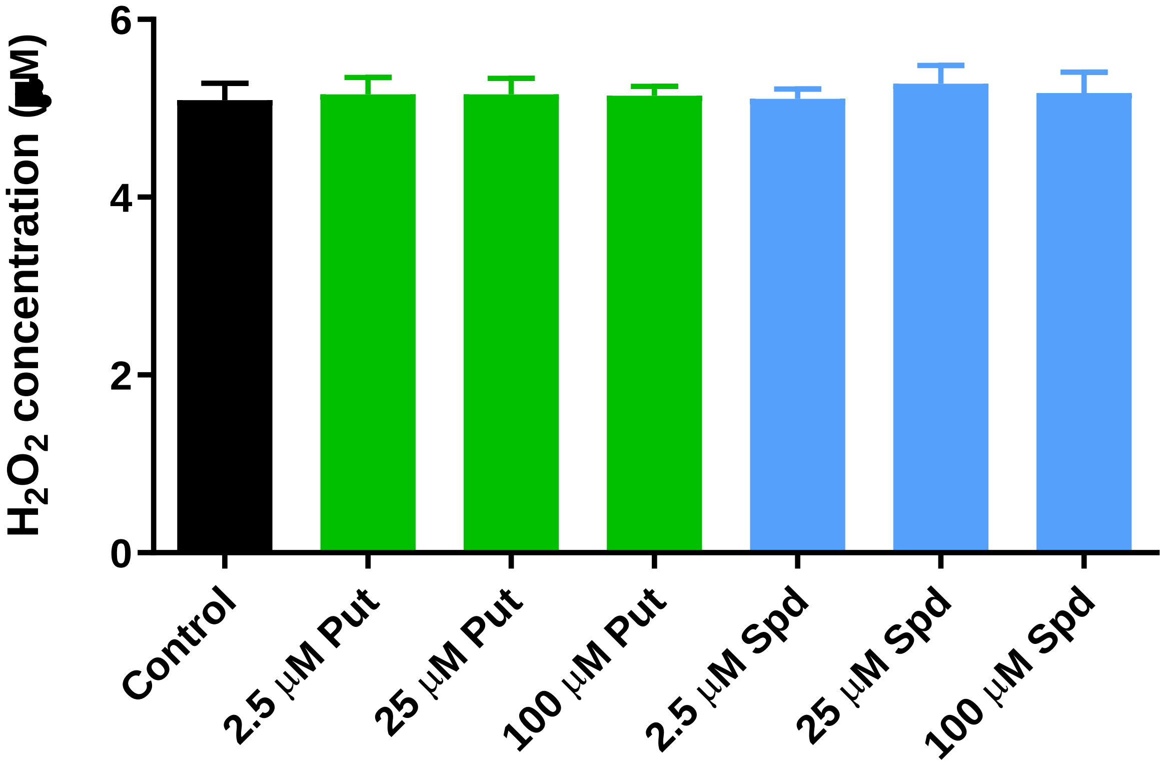

Figure S2. Quenching activity of polyamines on the oxidative effects of H_2O_2. Different concentrations of Put and Spd were incubated with 5 μM H_2O_2 in the presence of Amplex Red and horseradish peroxidase for 15 minutes, and the fluorescence determined using excitation and emission wavelengths of 571 nm and 585 nm, respectively.
