# Supplementary material for: Polyamine-mediated mechanisms contribute to oxidative stress tolerance in Pseudomonas syringae
Source: Sci Rep. 2023 Mar 15;13:4279. doi: 10.1038/s41598-023-31239-x (PMC10017717; doi:10.1038/s41598-023-31239-x)
Supplement: Supplementary file 3 — Supplementary Figure S3. [file 41598_2023_31239_MOESM3_ESM.pdf]

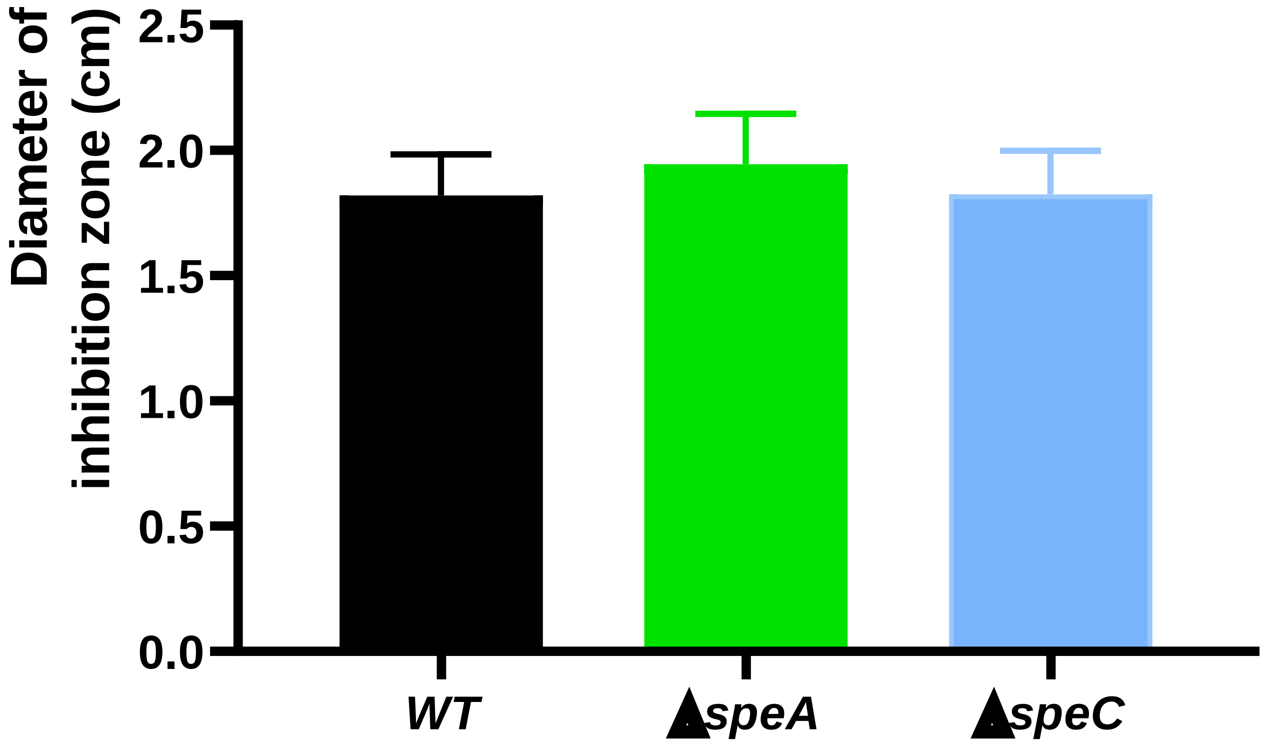

Figure S3. A. Bacterial growth inhibition in the presence of H_2O_2. Discs containing 10 µl of 30% H_2O_2 were placed on a lawn of the indicated bacterial strains in M9 agar. The diameters of the inhibition zones were determined after 48 h. Statistically significant differences on the diameters of inhibition zones were compared to WT using the Student´s t test.
