# Supplementary material for: Polyamine-mediated mechanisms contribute to oxidative stress tolerance in Pseudomonas syringae
Source: Sci Rep. 2023 Mar 15;13:4279. doi: 10.1038/s41598-023-31239-x (PMC10017717; doi:10.1038/s41598-023-31239-x)
Supplement: Supplementary file 4 — Supplementary Figure S4. [file 41598_2023_31239_MOESM4_ESM.pdf]

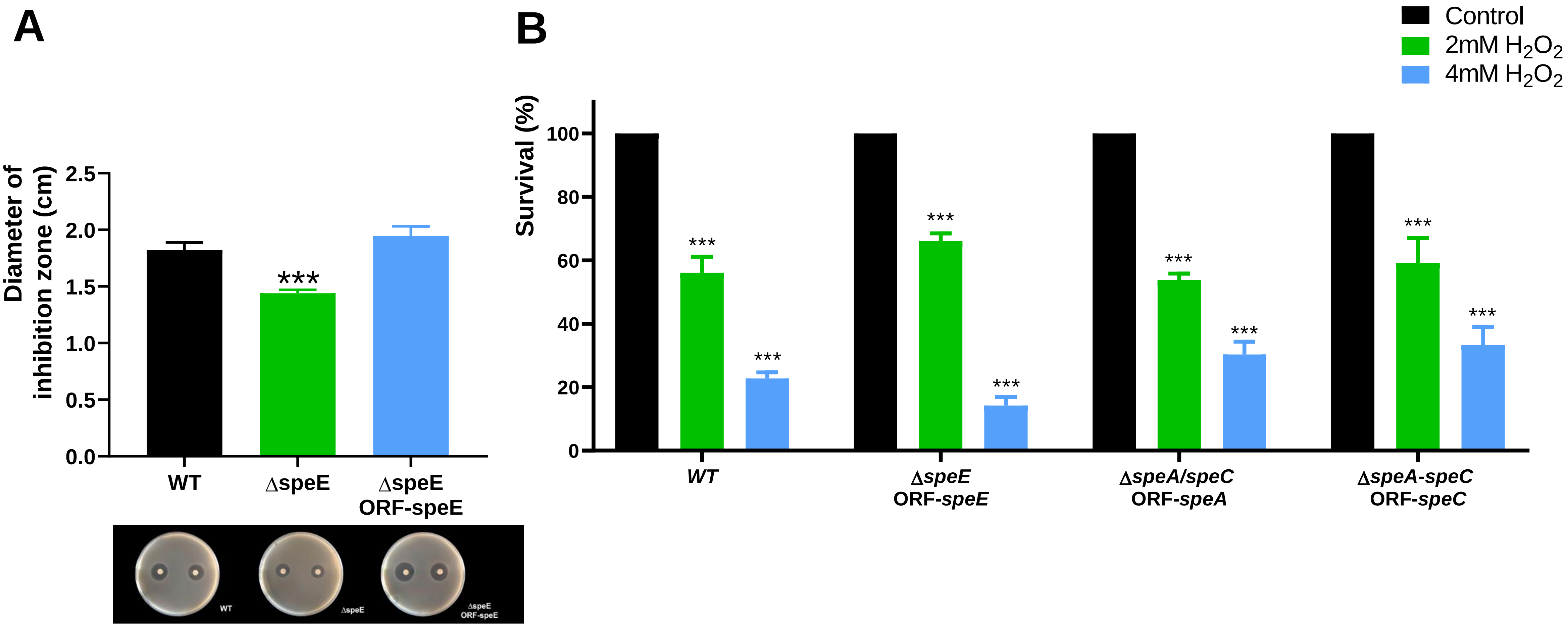

Figure S4. A. Bacterial growth inhibition in the presence of H_2O_2. Discs containing 10 µl of 30% H_2O_2 were placed on a lawn of the indicated bacterial strains in M9 agar. The diameters of the inhibition zones were determined after 48 h. Statistically significant differences on the diameters of inhibition zones were compared to WT using the Student´s t test. **B. Cell survival in the presence of H_2O_2.** Cultures of *Pst* DC3000 (WT) and reconstituted mutant strains in M9 (initial OD_{600}=0.2) were incubated for 1 h in under control conditions or in the presence of 2 or 4 mM H_2O_2. After this period, CFUs were determined and expressed as a percentage of the values quantified in control cultures (black bars). Statistically significant differences were evaluated by one-way ANOVA followed by Tukey´s multiple comparison tests. Asterisks on top of bars indicate differences between controls and H_2O_2-treated cells. ***=p<0,001
