# Supplementary material for: Polyamine-mediated mechanisms contribute to oxidative stress tolerance in Pseudomonas syringae
Source: Sci Rep. 2023 Mar 15;13:4279. doi: 10.1038/s41598-023-31239-x (PMC10017717; doi:10.1038/s41598-023-31239-x)
Supplement: Supplementary file 5 — Supplementary Figure S5. [file 41598_2023_31239_MOESM5_ESM.pdf]

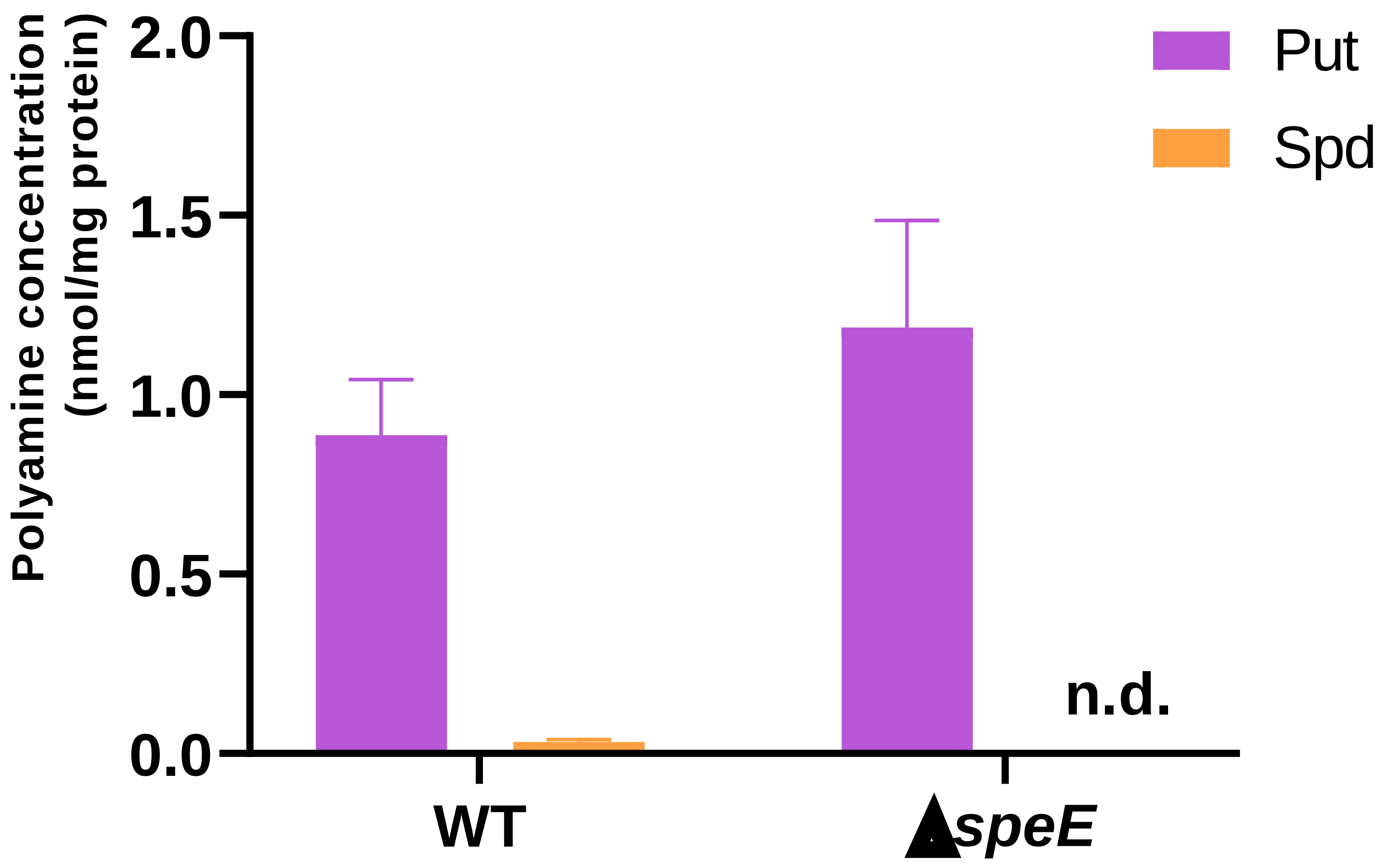

Figure S5. Membrane-bound polyamines in *Pst* DC3000. The WT and Δ*speE* strains were growth in M9 for 6h and isolated by centrifugation. Membrane-bound polyamines in samples were detached following the protocol optimized by Johnson *et al* (2012) and quantified as described in Materials and Methods. Concentrations of polyamines were compared to those obtained under control conditions using the Student´s t test n.d.=not detected.
