# Supplementary material for: Polyamine-mediated mechanisms contribute to oxidative stress tolerance in Pseudomonas syringae
Source: Sci Rep. 2023 Mar 15;13:4279. doi: 10.1038/s41598-023-31239-x (PMC10017717; doi:10.1038/s41598-023-31239-x)
Supplement: Supplementary file 6 — Supplementary Figure S6. [file 41598_2023_31239_MOESM6_ESM.pdf]

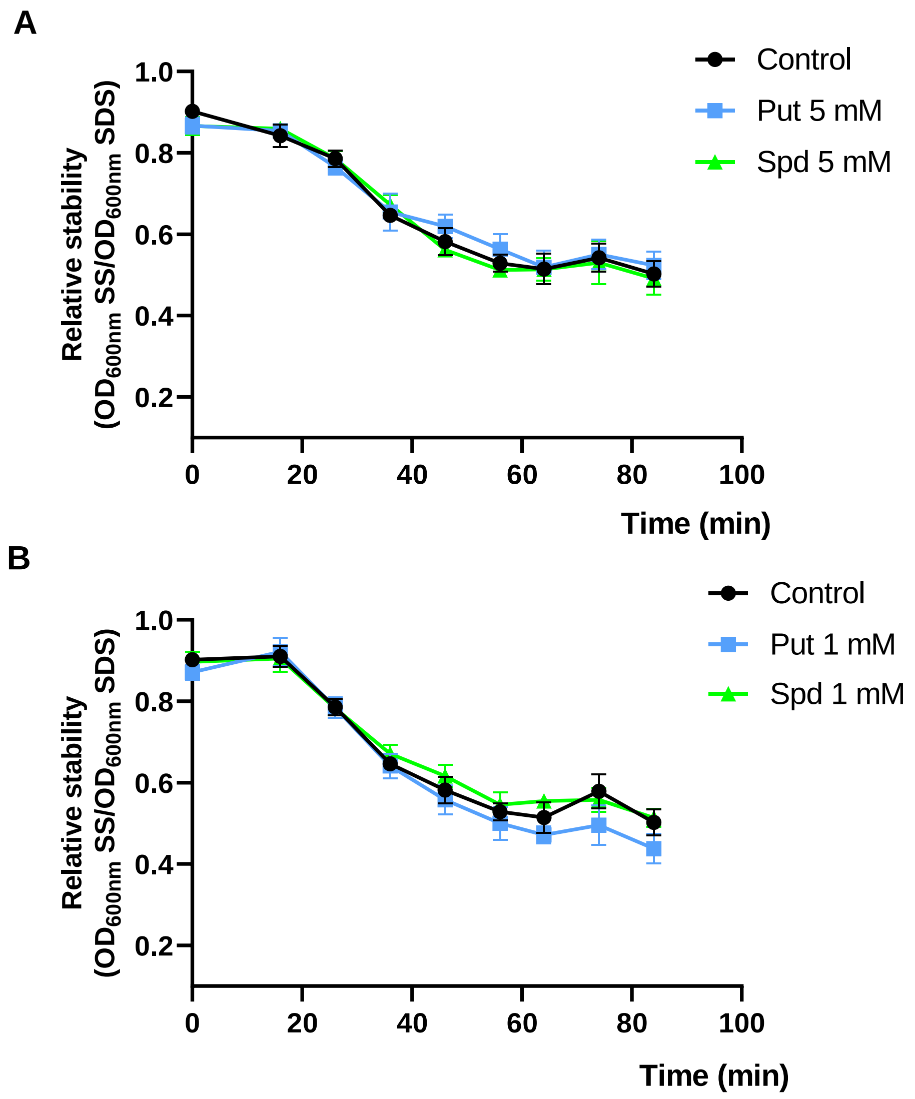

Figure S6. Effects of polyamines on the stability of the outer membrane. A. WT cells were incubated in Put or Spd 5 mM for 1 h before incubation in saline solution (SS) or 0,1% SDS (SDS), and the OD_{600} of the culture recorded during 60 min. Membrane stability is expressed as the absorbance in SDS relative to that in SS. **B.** The same approach as in **A** was used, but cells were previously treated with NaCl 1M to detached membrane-bound polyamines
