# Supplementary material for: Polyamine-mediated mechanisms contribute to oxidative stress tolerance in Pseudomonas syringae
Source: Sci Rep. 2023 Mar 15;13:4279. doi: 10.1038/s41598-023-31239-x (PMC10017717; doi:10.1038/s41598-023-31239-x)
Supplement: Supplementary file 7 — Supplementary Table S1. [file 41598_2023_31239_MOESM7_ESM.docx]

| **Table S1. Primers used in this work** | | | | | |
| --- | --- | --- | --- | --- | --- |
| **ID** | **Sequence** | | | **Restriction enzyme sites** | |
| speA-AF | | AACGGATCCGGGGACCTCTGTGTGCATCG | *BamH*I | |  |
| speA-AR | | TGCCCTCAGGACGACAAGTATGTGCGTCGTACGGACATCGAGG | - | |  |
| speA-BF | | ATGTCCGTACGACGCACATACTTGTCGTCCTGAGGGCACG | - | |  |
| speA-BR | | GCAAAGCTTAGCCCCTTCTCCAACTGCCT | *Hind*III | |  |
| speA-seqA | | GGATCGCTCAAGGACGATGT | - | |  |
| speA-seqB | | TCGGATTCAGCGAGTTCAGC | - | |  |
| speC-AF | | TAAGCAGAATTCCTCGCTGGTCAGCATCAC | *EcoR*I | |  |
| speC-AR | | AAAATTTACAGGTAGAACGAGGCGGTATCAATGACCACGA | - | |  |
| speC-BF | | TCGTGGTCATTGATACCGCCTCGTTCTACCTGTAAATTTTCAGC | - | |  |
| speC-BR | | CTGCAGGCAGGATGCGATGGTAATCAGGT | *Pst*I | |  |
| speC-seqA | | CAGGTCCTTGGGAAGGCA | - | |  |
| speC-seqB | | GCGGTTCAACTGAAGTAACC | - | |  |
| speE-AF | | TAAGCAGAATTCACCCGATTGATGCGCTCC | *EcoR*I | |  |
| speE-AR | | GTCTGTCAGTCGTTACTGGCCTGATAATCGCTCATGGGTAAGC | - | |  |
| speE-BF | | ATGAGCGATTATCAGGCCAGTAACGACTGACAGAC | - | |  |
| speE-BR | | CGAGTCGACTAACGCACCACCGGAAAGC | *Sal*I | |  |
| speE-seqA | | GGCGCAACTATCCTAGCA | - | |  |
| speE-seqB | | CCATTGAACAGCGCTTCGA | - | |  |
| GFPuv-F | | GGGGTACCAGTAAAGGAGAAGAACTTTTCAC | *Kpn*I | |  |
| PromKatB-F | | CGGAATTCCGACACTGTCATCTTCCTGC | *EcoR*I | |  |
| PromKatB-R | | GGGGTACCATGAACCATGTGTCTGGACC | *Kpn*I | |  |
| PromKatG-F | | CGGAATTCCGCATCAGTTCGATGACTGC | *EcoR*I | |  |
| PromKatG-R | | GGGGTACCCAGTTGACATGCTTGATACACC | *Kpn*I | |  |
| roGFP-F | | CCTCTAGATTTAAGAAGGAGATATACAT | *Xba*I | |  |
| roGFP-R | | GGCTGCAGTTATTTGTATAGTTCATCCATGCC | *Pst*I | |  |
| M13-F | | GTAAAACGACGGCCAGT | - | |  |
| M13-R | | CAGGAAACAGCTATGAC | - | |  |
| pK18M13-R | | AACAGCTATGACATGA | - | |  |
